# Drug-Related Genomics in Cancer and Immunological Diseases

**DOI:** 10.1155/2014/306980

**Published:** 2014-07-23

**Authors:** Ji-Fu Wei, Yong-Qing Wang, Huai-Rong Luo

**Affiliations:** ^1^Research Division of Clinical Pharmacology, The First Affiliated Hospital of Nanjing Medical University, 300 Guangzhou Road, Nanjing, Jiangsu 210029, China; ^2^State Key Laboratory of Phytochemistry and Plant Resources in West China, Kunming Institute of Botany, Chinese Academy of Sciences, Kunming, Yunnan 650201, China

Drug-related genes are defined as the genes which encode enzymes, transporters, and targets related to drug absorption, distribution, and metabolism to excretion. The studies on drug-related genomics in cancer and immunological diseases could enhance the understanding of the role of drug-related genes in the occurrence and development of cancer or immunological diseases. Pharmacogenomics research can help clinicians predict the efficacy of drugs in the body and avoid some side effects. In this respect, this special issue will add a few new points in the picture of drug-related genomics in cancer and immunological diseases.

C. Liu et al. in “*A promoter region polymorphism in PDCD-1 gene is associated with risk of rheumatoid arthritis in the Han Chinese population of Southeastern China*” provide an association between PDCD-1 polymorphism rs36084323 and rheumatoid arthritis (RA) risk. The potential mechanism might be related to the association between unregulated expressions of PDCD-1 on activated CD4+T cells in RA patients. They showed that the SNP rs36084323 could be a biomarker of early diagnosis of RA and a suitable indicator of utilizing PDCD -1 inhibitor for the treatment of RA.

In the study “*Upregulated PD-1 expression is associated with the development of systemic lupus erythematosus, but not the PD-1.1 allele of the PDCD1 gene,*” Q. Jiao et al. found that the PD-1 expression levels of systemic lupus erythematosus (SLE) patients were significantly increased compared with those of the healthy controls. Furthermore, the unregulated PD-1 expression levels in SLE patients were greatly associated with SLEDAI scores. These results suggest that increased expression of PD-1 may correlate with the pathogenesis of SLE, unregulated PD-1 expression may be a biomarker for SLE diagnosis, and PD-1 inhibitor may be useful for SLE treatment.

The association between cytokines and cytokine receptors and psoriasis is discussed in “*Genetic variations of cytokines and cytokine receptors in psoriasis patients from China*” by X.-L. Li et al. They genotyped seven SNPs in candidate genes of six ILs and only the rs3212227 in the IL12B gene was found to be associated with psoriasis at genotypic level in the studied population. Furthermore, the studied Chinese population has extremely low minor allele frequency for IL23R. Together, the author discussed the unique genetic patterns in the Chinese population that may be in part responsible for the lower risk for psoriasis in this population.

Nuclear factor-*κ*B was found to be associated with the pathogenesis of numerous malignancies. In the review article “*Association between NFKB1 −94ins/del ATTG promoter polymorphism and cancer susceptibility: an updated meta-analysis,*” X. Yang et al. showed that the SNP was significantly associated with cancer risk in four genetic models. The stratified analyses revealed that the polymorphism can exert race- and cancer-specific effects on cancer risk.

Polo-like kinase 1 (PLK1), one of serine/threonine-protein kinases, has been demonstrated to play pivotal roles in malignant transformation. “*High expression of polo-like kinase 1 is associated with early development of hepatocellular carcinoma*” by W. Sun et al. showed that expression of PLK1 was increased significantly in HCC tissues than that of corresponding normal liver tissues and PLK1 was related to the HCC cell differentiation or capsule invasion. Their results also indicated that the potential mechanisms of PLK1 inhibition regulated cell growth involving the dysregulated expression of caspase, Bcl, and p53. Therefore, the PLK1 expression might be an independent prognostic factor for HCC and targeting PLK1 might be a useful strategy for diagnosis and treatment of human HCC.

In the study “*Molecular evolution of the vertebrate FK506 binding protein 25,*” F. Liu et al. calculated the nonsynonymous and synonymous substitution rates, suggesting that FKBP25 undergoes purifying selection throughout the whole vertebrate evolution. Moreover, the result of site-specific tests showed that no sites were detected under positive selection. Only one PPIase domain was detected by searching FKBP25 sequences at Pfam and SMART domain databases. The result of this study suggests that the purifying selection triggers FKBP25 evolutionary history, which allows us to discover the complete role of the PPIase domain in the interaction between FKBP25 and nuclear proteins.

The house dust mites are major sources of indoor allergens for humans. X. Li et al. discussed the relation between Der f 25 and dermatophagoides farinae in “*In silico prediction of T and B cell epitopes of Der f 25 in dermatophagoides farinae.*” The sequence and structure analysis by them identified that Der f 25 belongs to the triosephosphate isomerase family and exhibited a triosephosphate isomerase pattern (PS001371). Eight B cell epitopes (11–18, 30–35, 71–77, 99–107, 132–138, 173–187, 193–197, and 211–224) and five T cell epitopes including 26–34, 38–54, 66–74, 142–151, and 239–247 were predicted in this study. These results can be used to benefit allergen immunotherapies and reduce the frequency of mite allergic reactions.

*Ji-Fu Wei* *Ji-Fu Wei* *Yong-Qing Wang* *Yong-Qing Wang* *Huai-Rong Luo* *Huai-Rong Luo*

